# A RE-AIM evaluation of Healthy Together: a family-centred program to support children’s healthy weights

**DOI:** 10.1186/s12889-020-09737-8

**Published:** 2020-11-23

**Authors:** Joan L. Bottorff, Anne Huisken, Michele Hopkins, Catherine Nesmith

**Affiliations:** 1 Institute for Healthy Living and Chronic Disease Prevention, University of British Columbia, 1147 Research Road, Art 223, Kelowna, BC V1V 1V7 Canada; 2 The Bridge Youth and Family Services, Kelowna, BC Canada

**Keywords:** Family health, Healthy lifestyles, Pediatric obesity, Obesity prevention, Healthy weights, Health promotion, Health behavior, Family-focused intervention, Parenting, Program evaluation

## Abstract

**Background:**

Healthy Together (HT) is family-centered program to support healthy eating and physical activity designed for implementation in community organizations serving families who may be experiencing vulnerabilities (e.g., related to low income, isolation, ethnicity, immigrant/refugee status, and/or Indigenous background). The purpose of this study was to conduct an evaluation of HT in a real-world, scale-up phase using the RE-AIM framework.

**Methods:**

Using a cross-sectional, non-comparative design, a community-based program evaluation was conducted in 29 organizations implementing HT as part of their core service programs. Data were collected using questionnaires with program participants and facilitators, and interviews with directors of participating organizations. Quantitative data were analyzed using descriptive statistics and qualitative data were content analyzed.

**Results:**

With regards to *Reach,* over 3400 caregivers, children and youth attended community programming that offered HT. Among those attending on the scheduled day for the evaluation, 663 completed the questionnaires. The majority of caregiver respondents (*n* = 431) were female (92%) and attended with children 0–6 years. Respondents also included children 4–6 years (*n* = 142) and 7–12 years (*n* = 65), and youth 13–18 years (*n* = 25). *Effectiveness* was demonstrated in reported improvements in physical activity, healthy eating, and strengthened social connections. HT was also widely supported by participants and facilitators. *Adoption* was influenced by the desire to enrich core service programs for families, HT’s fit within existing programs, organizational commitment, and funding support. *Implementation* experiences indicated that fidelity to the HT program was generally maintained, with some setting specific adaptations. *Maintenance* of HT was influenced by financial and non-financial resources within community organizations. Most organizations also introduced new initiatives to extend support for healthy eating and physical activity.

**Conclusion:**

Our findings indicate improvements in healthy eating and physical activity, and social connections among program participants, as well as efforts by community organizations to create environments to support healthy weights. HT was successfully delivered in “real-world” community settings across multiple contexts and with families with diverse backgrounds. This along with strategies to support program implementation and sustainability indicate that HT provides a model for other public health interventions to promote family health and wellbeing.

**Trial registration:**

ClincialTrials.gov NCT03550248. Registered May 25, 2018

**Supplementary Information:**

S**upplementary information** accompanies this paper at 10.1186/s12889-020-09737-8.

## Background

In North America it is estimated that 57% of American youth will develop obesity by the age of 35 [1] and 1.10 million Canadian children are predicted to be living with obesity by 2030 [2]. The lifelong consequences associated with childhood obesity and overweight are well studied, and are known to increase risks of adult obesity and a myriad of preventable chronic diseases including metabolic syndrome, cardiovascular disease, diabetes, cancer and poor psychosocial health [3, 4].

The importance of the family unit as an integral factor in children’s healthy development and in addressing modifiable lifestyle risk factors associated with childhood obesity has been well documented [5, 6]. Parents and/or family caregivers play a key role in structuring opportunities to develop positive physical activity and healthy eating behaviors that support the development of children’s healthy lifestyles and prevent overweight and obesity. Although age-specific community interventions such as the FAMILI program offered in Head Start centres to families with children aged 2–5 years [7] and the I-Cook after school program offered to families with children 9–10 years [8] present promising approaches to promoting healthy lifestyles, programs are needed that can address the needs of children of all ages including youth [9], and that can be offered across a range of community contexts.

As part a national initiative to promote healthy weights for children, Healthy Together (HT) was developed to engage families with children of all ages (0–18 years) to learn about healthy lifestyles through cooking, eating, and doing physical activity together to create the conditions for healthy weights and prevent weight-related chronic disease later in life. The commitment to family education is a defining feature of the program. This includes involving both children and caregivers in learning together, and the use of a family-centred approach in the design of the core program components. Community organizations serving families who may be experiencing vulnerabilities (e.g., related to low income, isolation, ethnicity, immigrant/refugee status, and/Indigenous background) provide important settings to promote healthy lifestyles and to extend the reach of family lifestyle programs. HT was, therefore, designed for flexible delivery of its core elements to enable implementation in varied community settings and to support tailoring to meet the needs of families and children of different ages. Details of program development are available elsewhere [10]. Initially, the HT program included 5 weekly sessions delivered by trained facilitators. Findings of a pre-post evaluation in 10 settings indicated that HT represented a feasible and promising program [11]. Compared to baseline, caregivers reported more confidence in cooking healthy meals following the HT program and in including children and youth in meal/snack preparation. Significant increases in children’s and youth’s knowledge of recommendations for daily physical activity, and confidence in eating fruits and vegetables were also observed. These findings were supported by facilitators and directors who reported HT had a positive impact on those who attended, and recommended that HT be strengthened by extending it over a longer period of time, improving program resources to enhance health literacy and including low cost, healthy food in cooking activities. In an expansion study to assess the feasibility of integrating the HT program into core services to support program sustainability, facilitators in the 16 participating organizations were encouraged to extend the 5-week program over as many weeks as may be needed. Although the results of this pilot provided additional support for the HT program and the potential for sustainability, facilitators did not extend the program beyond the 5 weekly sessions (Steinberg, 2016 “unpublished data”).

Based on these experiences, the HT program was revised to divide the original content and activities into smaller learning units for delivery over more weekly sessions, provide additional resources (e.g., more recipes for healthy snacks and extra physical activities), and enhance program resources (e.g., for low literacy and low English proficiency). The revised HT program included 30 sessions, available in both English and French, and in print and online versions (see http://healthy-together.ca/). On completion of this work, efforts began to scale-up HT in community settings across Canada. The purpose of this study was to conduct an evaluation of the HT program in a real-world, scale-up phase using the RE-AIM framework.

RE-AIM is an evidence-based evaluation framework is that is commonly used to assess the real-world application and impact of public health interventions in communities [12]. The RE-AIM model includes five dimensions: *Reach* focuses on the characteristics of organizations and participants willing to participate in the intervention, *Effectiveness* is an assessment of how well the intervention achieves intended outcomes, *Adoption* assesses setting level influences that effect program initiation, *Implementation* focuses on the extent that the intervention is delivered as intended, and *Maintenance* measures the extent to which an intervention is maintained at the organizational and individual level.

## Methods

Using a cross-sectional, non-comparative design, a community-based program evaluation was conducted to assess the success of the implementation of HT across Canada. The study received ethical approval from the Behavioural Research Ethics Board at the University of British Columbia (H17–01200).

### Community organizations

Participating community organizations (*n* = 29) were selected based on: 1) a mandate to provide programs and services to families who may be experiencing vulnerabilities, 2) interest in integrating a new programming to enrich core services for families, 3) staff availability to attend a training workshop and commitment of the organization to support trained staff in delivering HT, and 4) willingness to participate in the evaluation. The majority of community organizations (*n* = 23) were contracted to delivered 30 HT sessions, the remaining 6 delivered 24 sessions. Each participating organization identified 2 staff members to attend a 2-day facilitator training program (with all travel and accommodation costs covered by the project) and was provided $7500 CDN to support program delivery (e.g., purchase of food, and supplies such as sports equipment and small cooking appliances). Funding was not provided for staff salaries to deliver the program, since the expectation was that HT would be integrated into existing programs. Organizations serving immigrant and refugee families were provided additional funding (up to $800 CDN) to support translation services. Implementation took place between May 2017 and January 2020.

### Participant recruitment

Since participating community organizations were encouraged to integrate HT into their core service programming, they recruited parents, other caregivers, and children (0–18 years) to participate in HT through their regular programs using their usual channels of communication (e.g., social media, monthly calendars, word of mouth and posters).

### Healthy Together program

The aim of the HT program is to provide families with tools and resources to improve their knowledge and confidence in areas of healthy eating and physical activity as a pathway to promote healthy weights. In this family-centred program, each session engages children and their parents/caregivers in three core components: 1) a learning activity promoting healthy weights, 2) cooking and eating together, and 3) physical activity. Age-based adaptions for each of the components are included, and a flexible approach to program delivery is supported. Trained HT facilitators delivering the program in interactive, group-based sessions are encouraged to adapt program activities to suit local settings and participant needs/preferences, as well as to enable integration with other programs offered within participating organizations. Based on youth preferences for a group program with their peers, HT program content for this age-group is designed to be family focused (e.g., engaging youth in cooking activities and types of physical activity they are encouraged to do with their families following the HT session) and delivered only to youth. At the organization level, program assistants support facilitators in the delivery of HT, and administrative oversight is provided by directors of the participating organization. Community organizations were also encouraged to use the HT program as a platform to advocate for changes in policies and practices within their organizations and in the broader community to create environments to support family healthy lifestyles.

### Data collection

The measures and data sources used in this evaluation for each of the dimensions of the RE-AIM framework [12] are shown in Table 1. The surveys were specifically developed for this study by the evaluation team in consultation with HT program leads (See Additional files 1, 2, 3, 4 and 5). Staff at participating organizations invited HT participants to complete surveys to provide indicators of program outcomes following the first and second set of 15 sessions, or the 24th session. Children and youth were also invited to complete short, age-appropriate surveys, and caregivers reported outcomes for their children under 4 years of age. Although surveys were available in English and French, translation services were provided to support participation of non-English/French speakers. Information about the study was included in a cover page to the survey; completion and return of the surveys by youth and caregiver participants implied consent. Signed assent from caregivers was obtained for all children between 4 and 12 years old.

**Table 1:** RE-AIM Evaluation of Healthy Together

RE-AIM Dimensions	Measures	Data Sources
**REACH**		
To what extent did HT reach the target population?	*Organization level:* • Types of families served by participating organizations *Individual level:* • Estimated attendance at HT • Extent to which HT participants reflect target population	• Director interviews • HT implementation summary • Survey data (participants) • Census data
**EFFECTIVENESS**		
How well did HT program achieve intended outcomes?	• Knowledge of healthy eating and physical activity as a result of HT (caregivers) • Healthy eating and physical activity behavior as a result of HT (caregivers, youth, children 7–12 yrs) • Perceived impact on wellbeing (caregivers, youth, children 7–12 yrs) • Perceived impact on social connections within families and community (caregivers, youth) • Perceptions of HT acceptability (caregivers, youth, children 7–12 yrs) • Facilitator feedback on HT program	• Participant surveys • HT Facilitator surveys
**ADOPTION**		
What setting level factors influenced initiation and integration HT into core service programs?	• Enablers and challenges to initiating/ integrating HT in core service programs • Perceptions of ease of using the program tools and resources to implement HT	• Facilitator survey • Director interviews
**IMPLEMENTATION**		
To what extent was HT delivered as intended?	• Consistency related to delivering core components of HT in core services • Factors influencing ability to implement HT program as intended • Types of adaptations for settings and to meet needs of participants	• Facilitator surveys • Director interviews • Implementation summary
**MAINTAINANCE**		
To what extent are organizations continuing to offer HT? How has offering HT influenced policy and practice?	• Number of organizations continuing or with plans to continue offering HT • Enablers and challenges to HT program maintenance at 6-month follow-up • Policy and/or practice changes to support healthy lifestyles because of HT	• Director interviews • Implementation summary

HT facilitators completed program evaluation forms and an implementation summary following delivery of 15 and 30 HT sessions, or at the end of the program when 24 sessions were offered. Each community organization had at least one trained facilitator to fill out the evaluation form.

Directors were invited to participate in two brief semi-structured telephone interviews after their organization implemented the first set of 15 HT sessions, and 6 months after delivering the second set of 15 HT sessions. For organizations implementing 24 sessions, directors were invited to participate in an interview after all 24 sessions were delivered and at a 6-month follow-up. Following informed consent, a semi-structured telephone interview was conducted, recorded and transcribed verbatim.

### Measures

#### Demographic characteristics

Demographic questions for caregivers and youth included age, sex, country of birth, and Indigenous identity. Children 4–6 and 7–12 years reported on age and sex. Attendance at HT sessions were gathered via self-report.

#### Caregiver knowledge of healthy eating and physical activity

Caregivers were asked if, as a result of HT, they learned about healthy food for their family, where to get healthy, low cost food, and how to plan and make healthy low-cost meals for their families (yes/no/not sure). If they responded “yes” to learning about healthy food, they were asked to list two things they learned. Caregivers were also asked to rate how helpful the HT program was in teaching them about physical activity for their family (1 = very helpful to 3 = not very helpful).

#### Healthy eating

Caregivers and youth reported on changes in frequency of sugary drink consumption and fruits and vegetable intake because of coming to HT using a 5-point Likert scale (1 = less often to 5 = more often). Caregivers were also asked what changes, if any, they made in purchasing healthy, low-cost foods and preparing healthy meals for their family.

Children 7–12 years were asked if, because of coming to HT, they ate fruits and vegetables everyday, a few times a week, a few times a month, or not at all. Children 4–6 years were simply asked to list the types of fruits and vegetable they ate the day before.

#### Physical activity

Caregivers and youth responded to the question, “How physically active are you now than you were before the program?” using a 5-point Likert scale (1 = much less active to 5 = much more active). Children 7–12 years were asked if they were more active because of coming to HT (everyday, a few times a week, a few times a month, not at all). Children 4–6 years were simply asked: “Can you tell me something you do with your family that makes your heart beat really fast?”

#### Perceived impact on wellbeing

In a multiple response question, caregivers and youth were asked: What changes did you notice as a result of coming to Healthy Together? For caregivers the response categories included feeling healthier, happier, having more fun with their families, feeling less stressed, improving their English, and no change. For youth response categories included feeling healthier, happier, less stressed, and no change.

#### Social connections

Caregivers and youth reported on the effectiveness of HT in improving relationships with their family, helping them make friends with other participants, learning about community resources and feeling connected to their community (1 = not effective to 5 = very effective).

#### Program acceptability

Caregivers and youth reported on perceived usefulness of the program using the following response options: very useful, somewhat useful, a little useful, or not very useful. Caregivers, youth, and children 7–12 years were asked if they would refer others/their friends to the HT program (yes/no/not sure). Children 4–6 years were asked if they would like to come to HT again (yes/no/not sure).

#### Facilitator feedback

Using Likert scales and open-ended questions, facilitators reported on HT with respect to: achieving program outcomes, the usefulness of program content for participants, implementation experiences, program adaptations, whether they would recommend HT as a core service, and additional resources required to deliver the HT at their organizations.

#### Director interviews

Two semi-structured telephone interviews were conducted with directors of participating organizations. The initial interview was conducted at the end of the first set of 15 sessions or at the end of 24 sessions in communities offering this format. The interview included questions about their experience of integrating HT within core service programs, how well HT met the needs of families, organizational support and resources required to deliver HT, and adaptations to the design or delivery of HT for their families/setting. Directors were also asked what organizational policies and practices, if any, were introduced as a result of offering HT.

The second interview with directors was held 6 months following the last HT session. This interview focused on the success and challenges of integrating HT as a core service program, and changes at the organization level that supported healthy lifestyles (e.g., new policies or practices because of offering HT). In addition, directors were asked about plans to maintain HT as an ongoing core service, and facilitators and barriers to continuing to offer HT. The mean duration of interviews was 31 min (range 17–60 min).

### Data analysis

Survey data were entered into SPSS (v.24) by trained research assistants and were cleaned and checked for errors using a double entry method. All Likert scale variables were recoded into three categories. For example, responses related to program effectiveness were recoded into: effective (4–5), neutral (3), and not effective (1–2). Data were analyzed using descriptive statistics. Digital recordings of interviews were transcribed verbatim by a trained research assistant. Open-ended responses and transcribed data were content analyzed by one of the authors (AH) and trained research assistants. A coding framework was developed inductively using low inference codes to enable a descriptive summary of the data [13]. Regular checks on consistency of coding were conducted. NVivo (v. 11) was used to facilitate retrieval and review of coded data.

## Results

### Reach

The HT program was offered to families receiving services provided by 29 community organizations in 7 provinces and 1 territory across Canada, and was offered a total 57 times across participating sites. The majority of sites offered HT as part of weekly drop-in programs. Fifteen organizations were located in small communities (population 1000–29,999), 2 organizations were located in medium-sized communities (population 30,000-99,999) and 12 organizations were located in larger urban centers (population 100,000 and over) [14]. Location influenced attendance patterns, with larger groups in urban centres (e.g., over 80 different caregivers and 100 different children aged 0–6 years attended HT drop-in sessions in one city compared to a similar program offered in a rural centre with 8 different adults and 8 different children attending the program). Among the organizations offering HT for older children, lower levels of attendance were observed in programs for older age groups (e.g., the number of youth attending ranged from 4 to 20, where as the number of children in the two programs for 7–12 year olds was 22 and 42). Directors reported that the HT participants reflected the families accessing services at their organization, including those families who may be experiencing vulnerabilities.

A total of approximately 3400 caregivers, children and youth attended community programming that offered HT. Attendees in the final sessions were invited to participate in the evaluation. Of these attendees, 663 completed post-program evaluations. Respondents were included from all sites. On average 80% (range 33–100%) of caregivers present on evaluation day completed surveys. All youth who were present for the evaluation completed the survey, and in groups that included children 7–12 and 4–6 the average participation was 74% (range 25–100%), with the exception of 4 sites where facilitators did not invite children 4–6 to participate. The final study sample included 431 caregivers and 232 youth and children (see Table 2). Compared to 2016 Census data (CD), HT caregivers were more likely to be female (92% HT versus 50% CD) [15], born outside of Canada (35.3% HT vs 21.9% CD) [16] and Indigenous (First Nations, Metis, Inuit) (7.2% HT vs 4.9% CD) [16]. Across implementation sites participation rates appeared to be similar when compared to attendance patterns. Overall, in the 15 session programs 30% of respondents reported attending 6–10 sessions and 31% attended 11–15 sessions. In programs offering 24 sessions, 41% attended 7–12 sessions and 26% attended 13–24 sessions. Reported barriers to participating in the program included the cost of transportation or sometimes being turned away because the program was at capacity.

**Table 2:** Demographic characteristics of Healthy Together participants completing the post-program evaluation

	Caregivers (***n*** = 431)	Youth 13–18 years (***n*** = 25)	Children 7–12 years (***n*** = 65)	Children 4–6 years (***n*** = 142)	Children 0–3 years (***n*** = 390)
Mean age (%)	34.1 (8.2)	15.4 (±1.8)	8.63 (1.9)	4.5 (1.3)	2 (0.97)
Female (%)	396 (91.6)	13 (52)	33 (50.8)	76 (53.5)	184 (47.2)
Born outside Canada (%)	153 (35.5)	1 (4)	–	–	–
Indigenous (%)	31 (7.2)	3 (12)	–	–	–

*Note:* Data were not collected for children 0–12 years on variables born outside of Canada and Indigenous. Caregivers reported on age and sex of children 0–3 years

Program evaluations were completed by 93 trained facilitators. As employees of the participating organizations, they all had experience in facilitating groups and working with families. A few facilitators were professionally trained as dietitians (*n* = 2) or physical education specialists (*n* = 2). Interviews were completed by directors of 27 of the 29 participating organizations; one director declined the invitation to participate and the second did not respond to invitations.

### Effectiveness

Program effectiveness was assessed based on perceptions related to the program outcomes by participants. In addition, since HT was delivered across multiple, real-world community settings with families with varied backgrounds and children of different ages, perceptions related to program acceptability were also obtained.

#### Caregiver knowledge of healthy eating and physical activity

At post-program, caregivers reported that HT helped them learn about healthy food for their family (*n* = 340, 78.9%), where to buy healthy low-cost food (*n* = 268, 62.1%), and how to plan and make healthy low-cost meals for their family (*n* = 330, 76.6%). Examples of what they learned about health eating included topics related to nutrition (e.g., sugar intake, reading food labels), meal preparation (healthy substitutions, benefits of involving family in meal preparation), and exposure to new foods. Caregivers also reported that HT was helpful in teaching them about physical activities for their family (*n* = 391, 86.1%).

#### Changes health eating and physical activity behavior

Self-reported changes in healthy eating and physical activity for caregivers, youth, and children 7–12 years of age are reported in Table 3. Most caregivers tried new things to promote a healthy lifestyle (*n* = 376, 87.3%). Commonly reported activities were involving their children in food decisions (e.g., meal preparation, shopping), choosing healthier food options, and planning their family schedules to include healthy meals and family-oriented physical activity.

**Table 3:** Assessments of Healthy Together program outcomes by caregivers (*n* = 431), youth (*n* = 25) and children 7–12 years old (*n* = 65)

Lifestyle behaviors because of Healthy Together…	Increased n(%)	About the same n(%)	Decreased n(%)	Missing n(%)
Caregivers: my family fruit and vegetable intake	267 (61.9)	142 (32.9)	5 (1.2)	17 (4)
Caregivers: my family sugary drink intake	13 (3)	163 (37.8)	234 (54.3)	21 (4.9)
Caregivers: my physical activity level	189 (44.3)	216 (50.1)	15 (3.5)	11 (2.6)
Caregivers: my children’s physical activity level	220 (51.1)	181 (42)	11 (2.6)	19 (4.4)
Youth: my fruit and vegetable intake	22 (88)	3(12)	0 (0)	0 (0)
Youth: my sugary drink intake	1 (4)	4 (16)	20 (80)	0(0)
Youth: my physical activity level	16 (64)	5 (20)	1 (4)	3 (12)
	Everyday	A few times a week	A few times a month/ Not at all	Missing
Children: I am more active	40 (61.5)	13 (20)	5 (7.7)	7 (10.8)
Children: I eat more fruits and vegetables	31 (56.9)	16 (24.6)	4 (6.2)	8 (12.3)
**SOCIAL CONNECTIONS** **How effective was Healthy Together in…**	**Effective**	**Neutral**	**Not effective**	**Missing**
Caregivers: helping develop better family relationships	241 (55.9)	119 (27.6)	52 (12.1)	19 (4.4)
Caregivers: helping make friends	302 (70.1)	75 (17.4)	36 (8.3)	18 (4.2)
Caregivers: learning about community resources/places	281 (65.2)	91 (21.1)	38 (8.8)	21 (4.9)
Caregiver: helping connect me with my community	282(65.4)	89 (20.6)	44 (10.2)	16 (3.7)
Youth: helping develop better family relationships	10 (40)	8 (32)	6 (24)	1 (4)
Youth: helping make friends	12 (48)	3 (12)	8 (32)	2 (8)
Youth: learning about community resources/places to meet my needs	13 (52)	8 (32)	3 (12)	1 (4)
Youth: helping connect me with my community	16 (64)	4 (16)	4 (16)	1 (4)

The majority of youth and children 7–12 years old reported helping prepare meals everyday or a few times a week. Children 4–6 years were able to identify types of physical activity that increased their heartbeat (e.g., running) and, on average, listed at least three fruits or vegetables they ate on the previous day. Most children in this group also reported they helped prepare meals with their families (*n* = 122, 85.9%).

#### Perceived impact on social connections within families and communities

The majority of caregivers and youth reported that HT was effective in supporting social connections (see Table 3). In addition, more than a third of caregivers (*n* = 184, 42.7%) reported using services or attending programs in their community that they had learned about in HT.

#### Perceived impact on wellbeing

Many caregivers and youth perceived that HT positively affected their wellbeing. Specifically, caregivers reported that they felt healthier (64%), were having more fun with their families (62.3%), felt happier (56.6%) and felt less stressed (43.2%). They also indicated that program helped them improve their English (18.7%). Youth also reported they felt healthier (83.3%), happier (66.7%), and less stressed (62.5%). Very few caregivers and youth reported no changes in their wellbeing.

#### Program acceptability

Responses across all participant groups were positive. The majority of caregivers reported that HT sessions were somewhat or very useful to them (81.6%) and that they would refer others to the program (82.8%). Examples of aspects of HT they valued included the practical tools and tips in HT, experiences with involving children in food preparation, and the opportunity that HT provided for a “safe place” for their children to play and interact with others. Caregivers suggested that more options for low-cost recipes and activities be included in the program.

Most youth reported the HT sessions were useful to them (84%), and they would refer their friends to the program (84%). They liked attending the program with their friends, the range of activities included in HT, learning to cook, and feeling included in the group. Some thought HT should be offered more than once a week. The majority of children 7–12 years (83.1%) and 4–6 years (92.9%) reported they would like to come to the program again.

Directors and facilitators perceived that HT’s group-based format fostered a cohesive and supportive environment for learning. They observed positive changes in caregiver engagement and participation in group-based activities, improved parent-child caregiver interactions, and increased social support among caregivers attending HT. Directors also observed that for youth the group dynamics and opportunity to learn with peers were particularly beneficial. In one group, the director described the youth as *“one big family”* because of participating in HT.

The majority of facilitators perceived that HT was effective in achieving program outcomes; specifically, indicating that HT increased participant knowledge about healthy eating (*n* = 86, 92.5%), improved nutrition/healthy eating (*n* = 68, 74.2%), increased knowledge of physical activity (*n* = 73, 78.5%), promoted physical activity (*n* = 72, 79.1%), improved child-caregiver interactions (*n* = 70, 75.2%), increased social support for families (*n* = 65, 69.9%), and linked families to other community resources or services (*n* = 57, 61.3%). Suggested improvements included reflecting cultural diversity in program resources, including more age-appropriate recipes and physical activities, and adapting more of the program resources for low literacy families.

### Adoption

Organizations that adopted the HT program included 24 providing programs to families with children 0–6 years, two organizations offering after school programming to children 7–12 years of age, and 3 organizations that provided programming to youth. In addition to community centres offering family-oriented services, sites included an immigrant service welcome centre, a youth detention centre, and a boys and girls club. Across all settings, program adoption was primarily motivated by the desire to enrich and expand programming for families/children. In this context, HT offered a “ready-to-use” program toolkit and web-based resources, funded facilitator training for their staff, and a flexible, adaptable program delivery model that supported integration into existing services. As such, organizations with enthusiastic directors and facilitators adopted the program even when they did not have ready access to space and resources to offer the full range of HT activities. For example, some delivered HT with limited access to kitchen space and equipment by focusing on assembling healthy snacks using non-cooking recipes. For others, the funding provided to implement HT enabled the purchase of sports equipment, food and small cooking appliances to support program delivery. In this respect, in budget constrained community organizations, the offer of modest funding also supported program adoption.

Nevertheless, successful adoption of HT required a commitment of organizational resources. All but 7 organizations enlisted regular program staff and/or volunteers to assist trained facilitators with delivery of HT. Although some directors observed that preparation was more time consuming for HT compared to other programs, staff became more efficient at planning sessions over time. When fluctuations in weekly drop-in attendance made it difficult to ensure sufficient groceries and supplies for each session, facilitators began to ask families to pre-register for weekly sessions. Implementation challenges were also related to unexpected staff shortages, and competing roles and responsibilities on the part of facilitators. In a few sites, adoption appeared to be primarily influenced by the organization’s efforts to sustain programming in the context of unstable core funding. For example, despite successfully offering the first 15 HT sessions, one organization was unable to deliver the remaining 15 sessions when their core funding collapsed and all programs were terminated.

### Implementation

Although program fidelity was not directly measured, based on facilitator implementation reports the majority of sites delivered the 3 core components of HT in each session. Modifications in relation to how these components were delivered were sometimes observed. For example, two sites alternated between the delivery of a cooking activity and a physical activity on a weekly basis to accommodate time needed to prepare a family-style meal and to incorporate field trips to recreation centers, outdoors spaces, or gyms.

Fidelity-inconsistent content modifications also observed. For instance, there were occasions when facilitators did not offer all 3 core components because supporting participants with immediate needs (e.g., related to family crisis, housing or food insecurity) took precedent. Integrating HT into a core service also meant that some facilitators had to prioritize the requirements of funded services. For example, facilitators who integrated HT into specifically funded programs (e.g., for prenatal and postpartum women) were mandated to cover a specific set of topics and HT did not always meet those requirements.

### Maintenance

Data related to maintenance was obtained from 23 of the 29 participating sites. Ongoing financial support was a key factor influencing sustainability of the HT program. In particular, directors recognized in comparison to other programs, providing food at each HT session increased program costs. Directors at 10 sites reported that they were actively looking for support to continue offering HT either through applications for external funding, or through the support of new or existing community partnerships.

Director responses describing organizational commitment to continue to offer the HT program were grouped into three categories: a) adopted HT as a core service and were offering the program at their organization at the time of the interview (*n* = 14), b) plans to offer HT in the next year (*n* = 6), and 3) no plans to continue to offer HT (*n* = 3). In the first category, organizations were motivated to continue to offer HT because of high levels of participant satisfaction and strong staff support for the program. When limited by available resources, the frequency of HT sessions was reduced (e.g., to biweekly or monthly) or HT was offered as an annual 30-week program. Only one director planned to train staff and expand HT to 9 additional sites in her region. In the second category, directors at 6 sites indicated they had plans in place to offer HT in some capacity over the next 12 months, although this was dependent on securing additional resources either through grants or in-kind donations. In the third category, directors at 3 sites had no plans to continue to offer HT due to financial constraints. Stagnant funding, financial restructuring, budget cuts, and grant-to-grant funding were put forward as reasons the organizations could not continue offering HT. Across all groups, continuing access to facilitator training was an identified need to address staff turnover and to train additional staff to support program expansion.

At 20 community organizations, HT inspired new health initiatives or policies that supported the promotion of healthy lifestyles. The most common initiatives focused on integrating nutrition education and physical activity into other programs, and new efforts to serve healthier snacks and beverages. For example, in one organization staff were making more foods “from scratch” because of HT. In another, the HT program inspired the introduction of a formalized healthy beverage policy and the inclusion of physical activity in all core service programs. Two organizations reported that because of HT they also made changes to improve staff wellness by introducing staff walking meetings and staff wellness days.

## Discussion

This study utilized the RE-AIM framework to evaluate the implementation of HT as part of core service programs in 29 community-based organizations serving vulnerable families. The findings highlight successes and real-world challenges, provide direction for supporting broad community-based implementation of HT to support healthy lifestyles for families, and offer insights for the field of community-based implementation science.

### Reach: did we reach the target group?

We were able to demonstrate the broad reach of HT. Our efforts to engage community-based organizations in diverse locations across Canada, with goals that were closely aligned with the aims of HT, was important in supporting the reach of HT. With well established mandates to provide services to vulnerable families, the 29 participating organizations were all well positioned to engage families in HT, thereby overcoming recruitment challenges that many public health interventions face. Also, the integration of HT into core service programs provided an important avenue to introduce families to HT within a familiar context and by trusted providers, and may be a key factor in engaging vulnerable families. This is supported, in part, by the strong participation rates across implementation sites even though HT was most often offered as part of drop-in programs. Of particular note is that immigrant and Indigenous families were well represented among HT participants, although they have been reported to be under-represented in most other family-based childhood obesity interventions [9]. That organizations were successful in engaging these families in HT is an important observation given that there is evidence that that immigrant children are at an increased risk of overweight and obesity after arriving in Canada [17], and that Indigenous children are at higher risk for obesity and overweight than other Canadian children [18].

Youth and male caregivers were under-represented groups among HT participants. Although only 3 community organizations offered HT to youth, their success in engaging vulnerable youth (e.g., youth detention centre, low income neighbourhood) indicates the potential for expanding the reach of HT to youth in the future. In relation to male caregivers, despite efforts to engage this group, they represented less than 10% of the HT participant caregivers. Growing evidence suggests that fathers play an important role in shaping their children’s physical activity and dietary behaviors [19]. Given rapidly changing gender roles and father involvement in childcare, it is particularly important that public health interventions strengthen efforts to engage fathers [20]. Future research is needed to explore male caregiver preferences for family lifestyle programs and how the HT program, in particular, can be adapted to increase their participation.

### Effectiveness: was the intervention effective?

Effectiveness was based on self-reported program outcomes following participation in HT, and data indicated these were consistent with HT’s goal to promote healthful lifestyles. For example, caregivers reported using HT recipes at home, involving their families in meal preparation, structuring family time for play and physical activity, and enhanced social interactions within families and their communities. Comparable family-centered programs to engage families in obesity prevention have reported similar promising results. For example, after attending six biweekly sessions, caregivers involved in the *iCook 4-H Study* reported improvements in mealtime activities, improved family enjoyment during mealtimes and play, and improved social connections between family members and peers, and increased community capacity to support healthy lifestyle behaviors [8]. In the *FAMILI study,* findings supported promising improvements in physical activity outcomes in preschool children and increased caregiver self-efficacy to promote healthy eating and support their family’s physical activity [7]. Unlike these programs, with HT we were able to show similar outcomes with a program that was designed for delivery to families with children 0–18 years. In addition, these outcomes extended to community settings, where HT was integrated into services for families with limited English proficiency. Others have shown that blending health literacy within English as a second language curriculum is an effective way to improve knowledge of health behaviors while simultaneously improving English language proficiency [21].

### Adoption: what setting level factors influenced initiation and integration HT into core service programs?

HT was adopted by a diverse set of organizations that shared a commitment to serving vulnerable families. Across these organizations, perceptions that HT aligned closely with organizational goals and priorities, and included a flexible delivery model that enabled its fit with their day-to-day activities supported adoption. For most, HT provided an opportunity to augment programming to families with the availability of valued support (e.g., facilitator training, a comprehensive program toolkit and web-based resources, and funding to cover program delivery costs). These findings are supported by others who have recognized that when programs are well aligned with an organization’s overall mission and daily practices, include options that allow implementers some flexibility in program delivery, and outcomes are perceived beneficial, the program is more likely to be implemented successfully in real-world settings [22]. Also demonstrated in this study is the potential for adoption of programs like HT in a range of settings serving diverse families by encouraging integration in core programs, thereby drawing on existing relationships with families and the ability of knowledgeable, community-based facilitators to tailor program activities to meet family needs. It is important to note, however, that while the modest funding provided to deliver HT was a factor in adoption, it appeared that in settings motivated to sustain existing services in the context of financial instability, the funding for HT did not guarantee successful adoption. In the context of integrating HT into ongoing programs, HT had to be balanced with the competing demands of other programs, emergent family priorities, and unexpected staff turnover/shortages, along with the extra work involved with program preparation.

Although successful adoption was supported by the resourcefulness of organizations and the enthusiasm for offering HT, refinements to the program such as reducing the complexity of food preparation (e.g., including options for simple, healthy snacks), adding short, low-resource physical activities, and delivering program activities in bit-sized pieces over multiple sessions are strategies with the potential to enable adoption as an integrated program. In addition, an online HT facilitator training program has been developed and is now available to provide convenient ready access to training to address emergent staffing needs.

### Implementation: to what extent was HT delivered as intended?

HT was integrated into a variety of core service programs. Facilitator training and ready access to program and web-based resources likely supported adherence to the program. For interventions to fit into the context of the communities they are implemented into, adaptations are a common occurrence [23]. While the effect of adaptions to the HT program on outcomes was not assessed, they were generally viewed as useful strategies made by experienced facilitators to maximize fit and successful integration of HT into existing core services in vastly different contexts while meeting the diverse needs of families. Nevertheless, since adaptation can also result in poorer outcomes, these findings point to the importance of continued efforts in scaling up family-centered interventions in community settings to characterize adaptations and contextual factors that are associated with achieving program goals and outcomes [24].

### Maintenance: to what extent are organizations continuing to offer HT? How has offering HT influenced policy and practice?

Promoting the integration of HT into core-service programs in community organizations operating on tight budgets was an effort to reduce costs associated with offering the program and promote sustainability. This strategy was successful with continuation plans in place or with concrete plans to offer the program in the future reported by 20 of the 29 participating organizations. By engaging existing staff and resources, organizations were well positioned to continue offering HT compared to the projected costs of offering a stand-alone program [11]. Nevertheless, continuing to offer HT was not cost neutral. Directors recognized this, pointing to financial constraints as main barriers to the long-term sustainability of the program. Albeit modest, additional funds were needed to purchase expendable resources including food to continue to offer HT. These findings indicate that in addition to developing health promotion programs that enable integration into core services, community organizations would benefit from tools to assist with securing additional funding and engaging partners. The development of regional and national partnerships to support the scale-up and sustainability of HT as a nation-wide initiative is also needed. In particular, strong support from reputable community organizations and an extensive network of community partnerships are factors that have shown to play a critical role in influencing long-term sustainability in other evidence-based programs [25]. The importance of supporting program sustainability should not be underestimated. Offering HT prompted changes in practices and policies to support healthy lifestyles within participating organizations, effectively creating environments that enabled healthy eating and physical activity for families and staff that extended well beyond the HT program. Promising is the potential to continue to expand these practices into other areas in the community to enable families in taking up healthy lifestyles.

### Strengths and limitations

Our findings need to be considered in light of limitations and the challenges in conducting community-based evaluations. In this study, a post-program evaluation design was used to reduce participant burden encountered in previous evaluations [11], and address the practical demands of involving families with children representing a wide range of ages in diverse settings. The surveys were available in French and English, and translators were provided by community organizations when this was needed, ensuring that all participants had an opportunity to complete data collection. Accordingly, our response rates were high among participants available to provide feedback on scheduled days for data collection (e.g., 80% for caregivers, 100% for youth, 74% for children 7–12 and 4–6 years). This success can be attributed to tailoring of study methods to support inclusion among all groups of participants (including young children), the collaborative relationships established with community partners, and the trusted environment of community settings where participation in the evaluation was invited. That said, our findings may not capture the views of participants that dropped out or did not attend the program frequently. These non-respondents may hold different opinions about the effectiveness and acceptability of the program. We also relied on self-reported measures. This approach, along with the use of age appropriate surveys that included modified questions and response options, may have inadvertently introduced a response bias and reduced the internal validity of these findings. In addition, although data were not collected to assess maintenance of behavior change as a result of HT, changes in organizational policies and practices provide a promising environment for enabling healthful behaviors of children and families who access the services provided by community organizations. Interviews with directors over time, including a 6-month interview following the last HT session, yielded important data on factors influencing maintenance of HT at the organizational level as well as indicators of success.

## Conclusion

The findings of this community-based evaluation indicate that HT was widely supported by participants, facilitators, and directors, and successfully delivered in “real-world” community settings across multiple contexts and with families with diverse backgrounds. Using the RE-AIM framework provided important insights about program strengths and areas for refinements to enhance overall quality. Based on the RE-AIM model, strengths of the HT program include: a) its potential for broad reach when delivered by community-based organizations, b) effective support for healthy lifestyles together with strengthening family interactions and social connections among families who may be experiencing vulnerabilities, c) enabling adoption and implementation across diverse settings with its program resources, facilitator training and flexible delivery model, and d) providing a foundation for practice and policy changes to create environments to maintain healthy eating and physical activity. The results of the RE-AIM evaluation also point to areas where HT could be strengthened, including developing strategies to support participation of under-represented groups (e.g., youth, male caregivers), program refinements to support implementation in busy, resourced constrained community organizations, and tools to enable program sustainability. Taken together the findings indicate that HT is a promising approach for achieving the goal of supporting healthy weights, and provides a model for other public health interventions to promote family health and wellbeing.

## Supplementary Information

**Additional file 1.**

**Additional file 2.**

**Additional file 3.**

**Additional file 4.**

**Additional file 5.**

## Data Availability

The datasets used and/or analysed during the current study are available from the corresponding author on reasonable request.

## Abbreviations

HT: Healthy Together
HWC: Healthy Weights for Children project
RE-AIM: Reach, Effectiveness, Adoption, Implementation, Maintenance
CD: Census data
CDN: Canadian dollar
