# Supplementary material for: A RE-AIM evaluation of Healthy Together: a family-centred program to support children’s healthy weights
Source: BMC Public Health. 2020 Nov 23;20:1754. doi: 10.1186/s12889-020-09737-8 (PMC7681950; doi:10.1186/s12889-020-09737-8)
Supplement: Supplementary file 1 — Additional file 1. [file 12889_2020_9737_MOESM1_ESM.docx]

**Parent/Caregiver/Guardian Cover Letter and Feedback Form**

**Healthy Together Children’s Health Program - Phase 3**

This letter is to invite your participation in an evaluation study of the *Healthy Together* program. The *Healthy Together* program offers a chance to learn about food and nutrition, physical activity and cooking. We wish to ask each person who comes to this program for their feedback, so we can make it better. We are inviting you to answer a few questions, if you would like to.

The questions are written out on a short questionnaire. Answering these questions is voluntary; you may choose to answer, or not answer, any of the questions. What you tell us about the program is important to us and will be used to improve the program. You are free to say anything about the program. It will take about 15 minutes to complete the questionnaire.

Answering the questions will not bring you any harm, or help you directly. However, your answers will help us learn how to improve the program for other families.

All information received will be confidential. Your name will not be included on the form. We will not be able to tell who has completed the form. All of the information collected will be securely stored at the University of British Columbia (Okanagan campus). No names will be included in any reports of this evaluation of *Healthy Together*.

If you have any questions about this project you may contact, Dr. Joan Bottorff at xxx-xxxx; [email address]. If you have any concerns about your rights or treatment as a research subject, please contact the Research Participant Complaint Line in the UBC Office of Research Services at xxx-xxxx or the UBC Okanagan Research Services Office at xxx-xxxx. It is also possible to contact the Research Participant Complaint Line by email [email address].

Completing the questionnaire confirms your agreement to participate and your understanding of this study.

Thank you in advance for helping us!

**Program Feedback Form for Parent/Caregivers/Guardians**

Please help us improve our program by taking 10-15 minutes to provide us with feedback. Answering the questions is voluntary; you may choose to answer or skip any of the questions. The information you provide is important and will be used to improve the program. Your examples are very helpful to us so we appreciate when you share your comments.

Please feel free to tell us anything about the program; we will not be able to tell who has completed this form.

Thank you for helping us!

1. How many sessions of ***Healthy Together*** did you attend (please circle your response):

| 1-5 sessions | 6-10 sessions | 11-15 sessions |
| --- | --- | --- |

1. Please tell us about your children that are participating in the ***Healthy Together*** program:

| **Child** | **Age (years/months)** | **Gender** |
| --- | --- | --- |
| #1 |  | 🞏 GIRL 🞏 BOY 🞏Prefer not to answer |
| #2 |  | 🞏 GIRL 🞏 BOY 🞏Prefer not to answer |
| #3 |  | 🞏 GIRL 🞏 BOY 🞏Prefer not to answer |
| #4 |  | 🞏 GIRL 🞏 BOY 🞏Prefer not to answer |

1. During the PAST week, how many days did you do the following kinds of **physical activity for more than 10 minutes**?

- Only count physical activity sessions that lasted **10 minutes or longer** in duration.
- If you have not performed any physical activity, please enter ‘0’ in that space.

| Type of Activity | How many days in the PAST week? | How many minutes per day? |
| --- | --- | --- |
| Vigorous activity (heart beats rapidly, sweating)  e.g., running, vigorous swimming, hockey, soccer, martial arts, bicycling long distances, aerobic dance classes, or other vigorous activities. |  |  |
| Moderate activity (tiring, light sweat)  e.g., fast walking, jogging, baseball, tennis, easy bicycling, easy swimming, dancing. |  |  |
| Mild physical activity (little effort, no sweat)  e.g., easy walking, yoga, golf, gardening |  |  |

1. How helpful was the ***Healthy Together*** program in teaching you about physical activities for your family?

(circle one)

| Very helpful | Somewhat helpful | Not very helpful |
| --- | --- | --- |

1. In your opinion, as a result of the ***Healthy Together*** program (Please **✓**one box to answer)

|  | Much less active  **1** | **2** | About the same  **3** | **4** | Much more active  **5** |
| --- | --- | --- | --- | --- | --- |
| How physically active are you now than you were before the program? |  |  |  |  |  |
| How physically active is your child (or children) now than they were before the program? |  |  |  |  |  |

1. As a family do you do more physical activity together now than before the ***Healthy Together*** program?

🞏 YES 🞏 NO 🞏 Not sure

If YES: Please give us an example of a physical activity that you now do together as a family.

|  |
| --- |

7. During the last week, how often did your child (children) spend time in front of a screen (e.g., watched TV/videos, used a tablet, computer/phone):

| _____ | # of days in past week | _______ | # of minutes per day | _____ | don’t know/not sure |
| --- | --- | --- | --- | --- | --- |

8. During the last week, how many servings of vegetables and fruits did your child (children) eat on a typical day? *(A serving is 1/2 cup of vegetables or juice, or 1 medium size fruit or vegetable)*

_______ number of servings per day

1. How often does your family do the following? (Please **✓**one box to answer)

|  | Every day | A few times a week | Few times a month | Not at all |
| --- | --- | --- | --- | --- |
| Consume **(or drink)** sugary drinks (e.g., fruit punch, pop, energy drink, slushies, chocolate milk) |  |  |  |  |
| Eat foods that are high in sodium (salt) |  |  |  |  |
| Eat whole grain foods (e.g., bread or cereal) |  |  |  |  |
| Eat fruit and vegetables |  |  |  |  |
| Help prepare meals or snacks |  |  |  |  |

1. In your opinion, as a result of the ***Healthy Together*** program how often does your family (Please **✓**one box to answer)

|  | Much less often  1 | 2 | About the same  3 | 4 | Much more often  5 |
| --- | --- | --- | --- | --- | --- |
| Consume (drink) sugary drinks? |  |  |  |  |  |
| Eat fruits and vegetables? |  |  |  |  |  |

1. As a result of coming to ***Healthy Together***, I learned about healthy food for my family.

🞏 YES 🞏 NO 🞏 Not sure

If YES, here are two things I learned about healthy food for my family:

| 1. |
| --- |
| 2. |

1. As a result of coming to ***Healthy Together***, I now plan and make healthy, low-cost meals for my family more often.

🞏 YES 🞏 NO 🞏 Not sure

1. As a result of coming to ***Healthy Together***, I know where to get healthy, low-cost food.

🞏 YES 🞏 NO 🞏 Not sure

If YES, here is an example of where I can get healthy low-cost food:

|  |
| --- |

1. Because of what I have learned in ***Healthy Together***, I have tried new things to promote a healthy lifestyle
   for my family:

🞏 Once or twice 🞏 Several times 🞏 Not at this time

| Please tell us something you have done with your child, children to promote healthier lifestyle. (e.g., prepare/choose healthier foods, get more physical activity, limit screen time, managing stress etc.) |
| --- |
| _____________________________________________________________________________________ |
| _____________________________________________________________________________________ |

1. In your opinion, how effective was the ***Healthy Together*** program in: (Please **✓**one box to answer)

|  | Not effective |  |  |  | Very effective |
| --- | --- | --- | --- | --- | --- |
|  | **1** | **2** | **3** | **4** | **5** |
| 1. Helping you develop better relationships with your family |  |  |  |  |  |
| 1. In helping you make friends with other parents or caregivers |  |  |  |  |  |
| 1. In learning about other resources or places in the community to meet my family’s needs |  |  |  |  |  |
| 1. In helping you feel connected with your community (meaning the people in your neighbourhood, town or city) |  |  |  |  |  |

1. As a result of the ***Healthy Together*** Program, I have shared the information that I learned in this program with other families?

🞏 YES 🞏 NO 🞏 Not sure

1. Have you used community resources or places that you have learned about in the ***Healthy Together*** program?

🞏 YES 🞏 NO 🞏 Not sure

If **YES**, please let us know about a resource or place in the community that you have used or visited.

|  |
| --- |
|  |

1. How easy was it for you to fit the information from the ***Healthy Together*** program into your culture?

| Very easy | Easy | Somewhat difficult | Very difficult | Does not apply |
| --- | --- | --- | --- | --- |

1. What changes have you noticed as a result of being in ***Healthy Together*?** (check all that apply)

| 🞏 I feel healthier | 🞏 I feel more comfortable meeting & talking with new people |
| --- | --- |
| 🞏 I am happier | 🞏 My English has improved |
| 🞏 I am less stressed | 🞏 I lost weight |
| 🞏 I saved money on groceries | 🞏 Other (specify)____________________________________ |
| 🞏 I am having more fun with my family | 🞏 No change |

1. Were the ***Healthy Together*** sessions useful to you? (circle one)

| Not very useful | A little useful | Somewhat useful | Very useful |
| --- | --- | --- | --- |

1. Were the ***Healthy Together*** sessions respectful of your culture? (circle one)

| Not very respectful | A little respectful | Somewhat respectful | Very respectful |
| --- | --- | --- | --- |

22. What did you like BEST about the ***Healthy Together*** program?

|  |
| --- |
|  |

1. What did you like LEAST about the ***Healthy Together*** program?

|  |
| --- |
|  |

1. What suggestions do you have to help us make the ***Healthy Together*** program better for other families?

|  |
| --- |
|  |

1. How can we get more fathers and/or male caregivers to come to the ***Healthy Together*** program?

|  |
| --- |
|  |
|  |

1. Having been through this program, would you refer others to ***Healthy Together***? (circle one)

| Definitely | Maybe | Probably not |
| --- | --- | --- |

1. Is there anything else you want to say about the ***Healthy Together*** program? If yes, please write it here.

|  |
| --- |
|  |
|  |
|  |
|  |

***Tell us a little about yourself:***

1. How old are you? ____________ years

29. Do you identify as:

🞏Female 🞏 Male 🞏 Transgender 🞏 Other, specify ________________ 🞏 Prefer not to answer

1. Were you born in Canada? 🞏Yes 🞏 No 🞏Prefer not to answer

If No, where were you born? ____________________________________

1. Do you identify as First Nations, Metis, or Inuit? 🞏Yes 🞏No 🞏Prefer not to answer

***Congratulations on being a promoter of healthier and more active lifestyle for your child!* Thank you** for providing your valuable feedback on the **Healthy Together** program.  If you have any questions or concerns about the questions you may contact [name].
