# Supplementary material for: A RE-AIM evaluation of Healthy Together: a family-centred program to support children’s healthy weights
Source: BMC Public Health. 2020 Nov 23;20:1754. doi: 10.1186/s12889-020-09737-8 (PMC7681950; doi:10.1186/s12889-020-09737-8)
Supplement: Supplementary file 2 — Additional file 2. [file 12889_2020_9737_MOESM2_ESM.docx]

**Healthy Together Children’s Health Program – Phase 3**

Parent/Caregiver or Guardian Letter

For Permission for Children’s (4-12yr) Participation

This letter is to invite your child’s participation in an evaluation of the *Healthy Togethe*r Program.

The *Healthy Together* program offers a chance to learn about food and nutrition, physical activity and cooking. We wish to ask each person who comes to this program for their feedback, so we can make it better. A child (4-12 years) in your care is participating in this program. We are asking your permission for your child to answer a few questions. Even if you agree, it is still your child’s choice whether or not he/she would like to answer the questions. Completing the questions tells us that your child has agreed to participate.

The questions are written out on a short questionnaire. Answering these questions is voluntary; your child may choose to answer, or not answer, any of the questions. What your child tells us about the program is important to us and will be used to improve the program. Your child is free to say anything about the program. It will take about 10-15 minutes to complete the questionnaire.

Answering the questions will not bring your child any harm, or help your child directly. However, your child’s answers will help us learn how to improve the program for other children and families.

All information received will be confidential. Your child’s name will not be included on the form. We will not be able to tell who has completed the form. All of the information collected will be securely stored at the University of British Columbia (Okanagan campus). No names will be included in any reports of this evaluation of *Healthy Together*.

If you have any questions about this project you may contact, Dr. Joan Bottorff at xxx-xxxx; [email address]. If you have any concerns about your child’s rights or treatment as a research subject, please contact the Research Participant Complaint Line in the UBC Office of Research Services at xxx-xxxx or the UBC Okanagan Research Services Office at xxx-xxx. It is also possible to contact the Research Participant Complaint Line by email [email address].

Your consent and your child’s participation is entirely voluntary. You may refuse to consent or withdraw your consent at any time without giving a reason and without penalty or consequence. Please sign below if you provide your consent for your child to be invited to participate in the evaluation of *Healthy Together*.

Signed: __________________________ Date:_____________

Child’s name: _____________________

Thank you for helping us!

**Healthy Together Questions for Children (4-6 years)**

***Note to Parents:*** We would like to know what your child thought of Healthy Together program. If you are willing to allow your child to provide feedback, please find a quiet place to ask your child the questions on this sheet. Please write their answers in the space provided. Your child is free to say anything about the program; we will not be able to tell who has completed this form. Thank you for helping us.

1. How many ***Heathy Together*** sessions did you come to?

| 1-5 sessions | 6-10 sessions | 11-15 sessions |
| --- | --- | --- |

1. Did you like coming here? YES NO NOT SURE
2. Can you tell me about something you do with your family that makes you move around a lot and makes your heart beat faster?

|  |  |
| --- | --- |
|  | |

4. Can you tell me all the fruits and vegetables you ate yesterday?

|  |
| --- |
|  |

1. Do you ever help make food with your family? YES NO
2. What is something that you learned from coming here?

|  |
| --- |
|  |

7. What is your MOST favourite part about coming here?

|  |
| --- |
|  |

1. What would you like to do more of when you come here?

|  |
| --- |
|  |

1. Would you like to come to this program again? YES NO NOT SURE

**Tell us a little about yourself:**

1. How old are you? ____________ years
2. Are you a …. GIRL BOY Prefer not to answer

**Congratulations on being a promoter of healthier and active lifestyle for children!
Thank you** for your help. If you have any questions or concerns about these questions, please contact [name].
