# Supplementary material for: A RE-AIM evaluation of Healthy Together: a family-centred program to support children’s healthy weights
Source: BMC Public Health. 2020 Nov 23;20:1754. doi: 10.1186/s12889-020-09737-8 (PMC7681950; doi:10.1186/s12889-020-09737-8)
Supplement: Supplementary file 3 — Additional file 3. [file 12889_2020_9737_MOESM3_ESM.docx]

**Healthy Together Children’s Health Program – Phase 3**

Parent/Caregiver or Guardian Letter

For Permission for Children’s (4-12yr) Participation

This letter is to invite your child’s participation in an evaluation of the *Healthy Togethe*r Program.

The *Healthy Together* program offers a chance to learn about food and nutrition, physical activity and cooking. We wish to ask each person who comes to this program for their feedback, so we can make it better. A child (4-12 years) in your care is participating in this program. We are asking your permission for your child to answer a few questions. Even if you agree, it is still your child’s choice whether or not he/she would like to answer the questions. Completing the questions tells us that your child has agreed to participate.

The questions are written out on a short questionnaire. Answering these questions is voluntary; your child may choose to answer, or not answer, any of the questions. What your child tells us about the program is important to us and will be used to improve the program. Your child is free to say anything about the program. It will take about 10-15 minutes to complete the questionnaire.

Answering the questions will not bring your child any harm, or help your child directly. However, your child’s answers will help us learn how to improve the program for other children and families.

All information received will be confidential. Your child’s name will not be included on the form. We will not be able to tell who has completed the form. All of the information collected will be securely stored at the University of British Columbia (Okanagan campus). No names will be included in any reports of this evaluation of *Healthy Together*.

If you have any questions about this project you may contact, Dr. Joan Bottorff at xxx-xxxx; [email address]. If you have any concerns about your child’s rights or treatment as a research subject, please contact the Research Participant Complaint Line in the UBC Office of Research Services at xxx-xxxx or the UBC Okanagan Research Services Office at xxx-xxx. It is also possible to contact the Research Participant Complaint Line by email [email address].

Your consent and your child’s participation is entirely voluntary. You may refuse to consent or withdraw your consent at any time without giving a reason and without penalty or consequence. Please sign below if you provide your consent for your child to be invited to participate in the evaluation of *Healthy Together*.

Signed: __________________________ Date:_____________

Child’s name: _____________________

Thank you for helping us!

**Children (7-12 years) Cover Letter and Feedback Form**

**Healthy Together Program – Phase 3 Evaluation**

Your parent or caregiver has said it to okay to invite you to help us learn about what people think about the *Healthy Together* program. However, it is still your choice about whether or not to take part. In the *Healthy Together* program people learn about food and nutrition, physical activity and cooking. We wish to ask each person who comes to this program for their feedback, so we can make it better. We are inviting you to answer a few questions, if you would like to.

The questions are written out on one page. Answering these questions is up to you. You may choose to answer, or not answer, any of the questions. What you tell us about the program is important to us and will be used to improve the program. You are free to say anything about the program. It will take about 10-15 minutes to do the questions.

Answering the questions will not bring you any harm, or help you directly. However, your answers will help us learn how to improve the program for other children like you.

All information we receive will be kept private. Your name will not be included on the form. We will not be able to tell who has completed the form.

If you or your parent/caregiver have any questions about this project you may contact, Dr. Joan Bottorff at xxx-xxxx; [[email](mailto:joan.bottorff@ubc.ca) address]. If you have any concerns about your rights or treatment as a research subject, please contact the Research Participant Complaint Line in the UBC Office of Research Services at xxx-xx xx or the UBC Okanagan Research Services Office at xxx-xxxx. It is also possible to contact the Research Participant Complaint Line by email [email address]

Completing the questions tells us that you agree to share your ideas and you know this is a study.

Thank you in advance for helping us!

**Healthy Together Program Feedback for Children (7-12y)**

We want you to tell us what you think about the **Healthy Together** program so we can make it better. You may choose to answer (or not answer) any of the questions. Please feel free to say anything you want to about the program. We will not be able to tell who has completed this form. Thank you for helping us!

1. How many sessions of ***Healthy Together*** did you attend (please circle your answer):

| 1-5 sessions | 6-10 sessions | 11-15 sessions |
| --- | --- | --- |

2. How much did you enjoy ***Healthy Together*** program? (Circle 1 face)


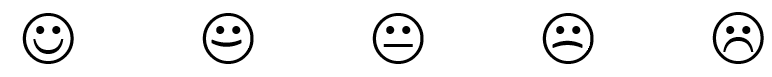


1. Because of coming to ***Healthy Together***… ( **✓**one box)

|  | Everyday | A few times a week | A few times a month | Not at all |
| --- | --- | --- | --- | --- |
| I am more active |  |  |  |  |
| I spend less time in front of screens (e.g.: TV, tablet, computer or phone) |  |  |  |  |
| I eat more vegetables and fruits |  |  |  |  |
| I help make meals with my family |  |  |  |  |
|  |  |  |  |  |

1. What is something that you learned in the ***Healthy Together*** program?

|  |
| --- |

1. What was your MOST favourite part of ***Healthy Together***?

|  |
| --- |

1. What did you like LEAST about ***Healthy Together***?

|  |
| --- |

7. Would you tell others to come to ***Healthy Together***? YES NO NOT SURE

**Tell us a little about yourself:**

1. How old are you? ____________ years
2. Are you a …. GIRL BOY Prefer not to answer

**Congratulations on being a promoter of healthier and active lifestyle!***Thank you for your help. If you have any questions or concerns about these questions, please contact [name]*
