# Supplementary material for: A RE-AIM evaluation of Healthy Together: a family-centred program to support children’s healthy weights
Source: BMC Public Health. 2020 Nov 23;20:1754. doi: 10.1186/s12889-020-09737-8 (PMC7681950; doi:10.1186/s12889-020-09737-8)
Supplement: Supplementary file 4 — Additional file 4. [file 12889_2020_9737_MOESM4_ESM.docx]

**Youth (13-18 years) Cover Letter and Feedback Form**

**Healthy Together Program – Phase 3 Evaluation**

This letter is to invite your participation in an evaluation study of the *Healthy Together* program. The *Healthy Together* program offers a chance to learn about food and nutrition, physical activity and cooking. We wish to ask each person who comes to this program for their feedback, so we can make it better. We are inviting you to answer a few questions, if you would like to.

The questions are written out on a short questionnaire. Answering these questions is voluntary. You may choose to answer, or not answer, any of the questions. What you tell us about the program is important to us and will be used to improve the program. You are free to say anything about the program. It will take about 10-15 minutes to complete the questions.

Answering the questions will not bring you any harm, or help you directly. However, your answers will help us learn how to improve the program for other youth like you.

All information we receive will be confidential (kept private). Your name will not be included on the questionnaire. We will not be able to tell who has completed the form. All of the information collected will be securely stored at the University of British Columbia (Okanagan campus). No names will be included in any reports of this evaluation of *Healthy Together*.

If you have any questions about this project you may contact, Dr. Joan Bottorff at xxx-xxxx; [email address]. If you have any concerns about your rights or treatment as a research subject, please contact the Research Participant Complaint Line in the UBC Office of Research Services at xxx-xxxx or the UBC Okanagan Research Services Office at xxx-xxxx. It is also possible to contact the Research Participant Complaint Line by email [email address].

Completing the questionnaire tells us that you agree to participate and you know this is an evaluation study.

Thank you in advance for helping us!

**Healthy Together Feedback Form for Youth (13-18y)**

Please help us make ‘**Healthy Together’** program better by answering these questions. Answering the questions is voluntary; you may choose to answer, or not answer, any of the questions. The information you provide is important and will be used to improve this program. Examples from your experience are very helpful so please take the time to write them. Please feel free to say anything you want to about the program; we will not be able to tell who has completed this form. Thank you for helping us!

1. How many sessions of ***Healthy Together*** did you attend (please circle your answer):

| 1-5 sessions | 6-10 sessions | 11-15 sessions |
| --- | --- | --- |

1. During the PAST week, how many days did you do the following kinds of **physical activity for more than 10 minutes**?

- Only count physical activity sessions that lasted **10 minutes or longer** in duration.
- If you have not performed any physical activity, please enter ‘0’ in that space.

| Type of Activity | How many days in the PAST week? | How many minutes per day? |
| --- | --- | --- |
| Vigorous activity (heart beats rapidly, sweating)  e.g., running, vigorous swimming, hockey, soccer, basketball, martial arts, long distance bicycling, aerobic dance classes, or other vigorous activities. |  |  |
| Moderate activity (tiring, light sweat)  e.g., fast walking, jogging, baseball, tennis, easy bicycling, easy swimming, skipping, dancing, or moderate play activities. |  |  |
| Mild physical activity (little effort, no sweat)  e.g., walking, yoga, playing catch, or other mild activities. |  |  |

1. In your opinion, as a result of the ***Healthy Together*** program, how physically active are you now than you were before the program? (circle one)

| Much less active |  | About the same |  | Much more active |
| --- | --- | --- | --- | --- |
| 1 | 2 | 3 | 4 | 5 |

4. During the last week how often did you spend time in front of a screen (e.g., watching TV/tablet, computer/phone):

|  | # of days |  | # of minutes per day |  | don’t know/not sure |
| --- | --- | --- | --- | --- | --- |

1. During the last week, about how many servings of fruit and vegetables did you have each day?

*(A serving is 1/2 cup of vegetables or juice, or 1 medium size fruit or vegetable)*

________ number of servings per day

1. How often do you do the following? (Please **✓**one box to answer)

|  | Every day | Few times a week | Few times a month | Not at all |
| --- | --- | --- | --- | --- |
| Consume **(or drink)** sugary drinks (fruit punch, pop, slushies, sports drink, energy drinks, specialty coffee, ice tea, bubble tea, chocolate milk, etc.) |  |  |  |  |
| Eat foods that are high in sodium (salt) |  |  |  |  |
| Eat whole grain foods (e.g., bread or cereal) |  |  |  |  |
| Eat fruit and vegetables |  |  |  |  |
| Help prepare meals |  |  |  |  |

1. In your opinion, as a result of the ***Healthy Together*** program how often do you:

(Please **✓**one box to answer)

|  | Much less often  **1** | **2** | About the same  **3** | **4** | Much more often  **5** |
| --- | --- | --- | --- | --- | --- |
| Consume (drink) sugary drinks? |  |  |  |  |  |
| Eat fruits and vegetables? |  |  |  |  |  |

1. In your opinion, how effective was the ***Healthy Together*** program in: (Please **✓**one box to answer)

|  | Not effective |  |  |  | Very effective |
| --- | --- | --- | --- | --- | --- |
|  | **1** | **2** | **3** | **4** | **5** |
| Helping you develop better relationships with your family |  |  |  |  |  |
| Helping you make friends |  |  |  |  |  |
| Learning about other resources or places in the community to meet your needs |  |  |  |  |  |

9. Have you used community resources or places that you have learned about in the ***Healthy Together*** program?

🞏 YES 🞏 NO 🞏 Not sure

If **YES**, please let us know about a resource or place in the community that you have used or visited.

|  |
| --- |
|  |

10. How effective was the ***Healthy Together*** program in helping you feel connected with your community (meaning the people in your class, school, neighbourhood, town or city). (circle one)

| Not Effective |  |  |  | Very effective |
| --- | --- | --- | --- | --- |
| **1** | **2** | **3** | **4** | **5** |

What is one thing you do that helps you feel connected with your community:

|  |
| --- |

1. What changes did you notice as a result of being in ***Healthy Together*?** (check all that apply)

| 🞏 I feel healthier | 🞏 Other (specify)_______________________________ |
| --- | --- |
| 🞏 I am happier | 🞏 No change |
| 🞏 I am less stressed |  |

1. Because of what I have learned in ***Healthy Together***, I have tried new things to promote a healthy lifestyle

for myself: (circle one)

| 3 or more times | 1 or 2 times | Not at this time |
| --- | --- | --- |

Tell us something you have done to promote a healthier lifestyle (e.g., prepare/choose healthier foods, get more physical activity, limit screen time, manage stress etc.)

|  |
| --- |
|  |

1. Were the ***Healthy Together*** sessions useful for you? (circle one)

| Not very useful | A little useful | Somewhat useful | Very useful |
| --- | --- | --- | --- |

1. Were the ***Healthy Together*** sessions respectful of your culture? (circle one)

| Not very respectful | A little respectful | Somewhat respectful | Very respectful |
| --- | --- | --- | --- |

1. What did you like BEST about the ***Healthy Together*** program?

|  |
| --- |
|  |

1. What did you like LEAST about the ***Healthy Together*** program?

|  |
| --- |
|  |

1. Having been through the ***Healthy Together*** program, would you tell your friends about it? (circle one)

| Definitely | Maybe | Probably not |
| --- | --- | --- |

1. Is there anything else you want to say about this program? If yes, please write it here.

|  |
| --- |
|  |
|  |
|  |

***Tell us a little about yourself:***19. How old are you? ____________ years
20. Do you identify as:
 🞏Female 🞏 Male 🞏 Transgender 🞏 Other, specify ________________ 🞏 Prefer not to answer
21. Were you born in Canada? 🞏Yes 🞏 No 🞏Prefer not to answer
 If No, where were you born? ____________________________________
22. Do you identify as First Nations, Metis, or Inuit? 🞏Yes 🞏No 🞏Prefer not to answer

***Congratulations on being a promoter of healthier and active lifestyle for youth!* THANK YOU** for providing your valuable feedback on our program.  If you have any questions or concerns about these questions, please contact [name]
