# Supplementary material for: A RE-AIM evaluation of Healthy Together: a family-centred program to support children’s healthy weights
Source: BMC Public Health. 2020 Nov 23;20:1754. doi: 10.1186/s12889-020-09737-8 (PMC7681950; doi:10.1186/s12889-020-09737-8)
Supplement: Supplementary file 5 — Additional file 5. [file 12889_2020_9737_MOESM5_ESM.docx]

**Healthy Together Facilitators - Cover Letter and Feedback Form**

**Healthy Together Children’s Health Program - Phase 3**

This letter is to invite your participation in an evaluation study of the *Healthy Together* Program. We wish to ask each person who is involved in implementing program for their feedback, so we can make it better. We are inviting you to answer a few questions, if you would like to.

The questions are written out on a short questionnaire. Answering these questions is voluntary; you may choose to answer, or not answer, any of the questions. What you tell us about the Healthy Together program is important to us and will be used to improve the program. You are free to say anything about the program. It will take about 10-15 minutes to complete the questionnaire.

Answering the questions will not bring you any harm, or help you directly. However, your answers will help us learn how to improve the program for other families.

All information received will be confidential. Your name will not be included on the form. We will not be able to tell who has completed the form. All of the information collected will be securely stored at the University of British Columbia (Okanagan campus). No names will be included in any reports of this evaluation of *Healthy Together*.

If you have any questions about this project you may contact, Dr. Joan Bottorff at xxx-xxxx; [email address]. If you have any concerns about your rights or treatment as a research subject, please contact the Research Participant Complaint Line in the UBC Office of Research Services at xxx-xxxx or the UBC Okanagan Research Services Office at xxx-xxxx. It is also possible to contact the Research Participant Complaint Line by email [email address].

Completing the questionnaire confirms your agreement to participate and your understanding of this study.

Thank you in advance for helping us!

**Program Feedback Form for Facilitators**

Please help us make **Healthy Together** better by taking a few minutes to answer some questions. Your answers are confidential and will not identify you.  Answering the questions is voluntary; you may choose to answer, or not answer, any of the questions. The information you provide is important and will be used to improve the program.

1. Based on your experience of delivering the **Healthy Together** program, how effective was the program in achieving the following outcomes for caregivers/children/youth. (Circle your answer)

|  | **Not**  **effective** |  |  |  | **Very effective** |
| --- | --- | --- | --- | --- | --- |
| 1. Improve child-caregiver interactions | 1 | 2 | 3 | 4 | 5 |
| 1. Increase knowledge of healthy eating | 1 | 2 | 3 | 4 | 5 |
| 1. Increase knowledge of physical activity | 1 | 2 | 3 | 4 | 5 |
| 1. Increase social support for families | 1 | 2 | 3 | 4 | 5 |
| 1. Link families to other resources or services | 1 | 2 | 3 | 4 | 5 |
| 1. Improve nutrition/healthy eating | 1 | 2 | 3 | 4 | 5 |
| 1. Promote physical activity | 1 | 2 | 3 | 4 | 5 |
| 1. Reduce screen time | 1 | 2 | 3 | 4 | 5 |

1. To what extent was the **Healthy Together** content relevant for promoting healthier lifestyles? (circle one)

| Very relevant | Somewhat relevant | Not very relevant | Not at all relevant |
| --- | --- | --- | --- |

1. To what extent was the content useful to participants?

| Very useful | Somewhat useful | Not very useful | Not at all useful |
| --- | --- | --- | --- |

1. To what extent did the **‘Healthy Together’ toolkit** adequately prepare you to deliver the program?

| Fully prepared me | Somewhat prepared me | Did not prepare me |
| --- | --- | --- |

1. How could the **Healthy Together toolkit** be improved?

|  |
| --- |
|  |
|  |

1. What did you like BEST about the **Healthy Together** program?

|  |
| --- |
|  |
|  |

1. What changes have you observed in the participants?

|  |
| --- |
|  |
|  |
|  |

1. Please describe any additional materials or information that you used to deliver the **Healthy Together** program:

|  |
| --- |
|  |
|  |
|  |

1. Please describe any adaptations or changes you made to the program.

|  |
| --- |
|  |
|  |
|  |

1. What were the benefits or positive results of the adaptations on the participants or the program?

|  |
| --- |
|  |
|  |

1. Have you continued to connect with families after the program?

🞏 YES 🞏 NO 🞏 Not sure

If YES, tell us how you have connected with families.

|  |
| --- |
|  |

1. Because of this program, have you made connections to professionals in the community?

🞏 YES 🞏 NO 🞏 Not sure

If YES, please list the agencies or professionals you have connected with.

|  |
| --- |
|  |
|  |

1. Based on your experience, would you recommend **Healthy Together** to your supervisors/managers as a core program within your organization?

🞏 YES 🞏 NO 🞏 Not sure

1. As a result of facilitating **Healthy Together**, what changes, if any, did you make or notice (check all that apply)

| 🞏 I increased my physical activity | | 🞏 I am less stressed |
| --- | --- | --- |
| 🞏 I increased my fruit & vegetable consumption | | 🞏 I shared information with my family |
| 🞏 I feel healthier | | 🞏 No change |
| 🞏 Other (specify) |  | |

1. Did you have a staff member or volunteer assist you with delivering the **Healthy Together** program?

🞏 YES 🞏 NO

If YES, what was their role in delivering the program? ____________________________________________

What challenges, if any, did they experience as a result of helping to deliver HT program?

|  |
| --- |
|  |
|  |

What successes did they have as a result of helping to deliver HT program?

|  |
| --- |
|  |
|  |

**Note:** If your helper staff person would like to offer separate feedback, please provide them with a blank copy of this questionnaire with the cover letter.

1. Please let us know if there is anything else you would like to share about the program

|  |
| --- |
|  |
|  |
|  |
|  |

**Thank you** for providing your valuable information for the **Healthy Together** program.  **Thank you** for your help. If you have any questions or concerns about these questions, please contact [name].
